# Mediating effects of physical fitness index between physical activity and executive function in Chinese adolescents: an observational study

**DOI:** 10.3389/fpsyg.2025.1592232

**Published:** 2025-04-25

**Authors:** Huipan Wu, Jinxian Wang, Jian Wu, Yuanyuan Ma, Yi Wang, Wenqing Duan

**Affiliations:** ^1^Research Center for Health Promotion of Children and Adolescents, Taiyuan Institute of Technology, Taiyuan, Shanxi, China; ^2^School of Sport and Physical Education, North University of China, Taiyuan, Shanxi, China; ^3^School of Sports Science, Jishou University, Jishou, Hunan, China

**Keywords:** physical fitness index, physical activity, inhibitory control, working memory, cognitive flexibility

## Abstract

**Objective:**

To explore the mediating effect of physical fitness index between physical activity and executive function in Chinese adolescents, and to provide a theoretical basis for improving adolescents’ physical fitness status and executive function.

**Methods:**

A total of 5,336 adolescents aged 13–18 years were sampled from September to December 2023 in Changzhi, Taizhou, Jishou, Nanchang, Suzhou, Xianyang, and Yulin, China, respectively, using stratified whole cluster random sampling. Adolescents’ physical activity, physical fitness index and executive function were assessed using questionnaires combined with tests. Descriptive analysis was used to describe the characteristics of each test index of adolescents, Pearson’s correlation was used to analyze the relationship between physical fitness index, physical activity, and executive function, and the mediation model was used to test the mediating effect of physical fitness index between physical activity and executive function.

**Results:**

The length of participation in moderate-to-vigorous physical activity was 110 (63, 187) min/week in the high fitness index group and 93 (53, 168) min/week in the low fitness index group, which was statistically different when comparing the two groups (Z = −4.286, *p* = 0.001); The response time for cognitive flexibility was (321.11 ± 142.79) milliseconds for females and (291.12 ± 137.09) milliseconds for males, which was statistically different when comparing the two groups (t = −7.816, *p* = 0.001). The correlation coefficient between moderate-to-vigorous physical activity and the physical fitness index was 0.039 (*p* < 0.05); the correlation coefficients between the physical fitness index and the 1-back response time, the 2-back response time, and the cognitive dexterity response time were −0.124, −0.180, and −0.100, respectively (all *p-*values < 0.05); There was no significant correlation between physical activity and any of the subfunctions of executive function (all *p*-values > 0.05). The mediation effect values of the physical fitness index between moderate-to-vigorous physical activity and 1-back response time, 2-back response time, and cognitive flexibility response time were −0.0007 (95% C1 = −0.0021 ~ −0.0003), −0.0013 (95% CI = −0.0039 ~ −0.0007), and −0.0004 (95% CI = − 0.0010 ~ −0.0002).

**Conclusion:**

Fully mediated effects of the adolescent fitness index between moderate-to-vigorous physical activity and working memory and cognitive flexibility.

## 1 Introduction

In recent decades, with the rapid development of the global economy and profound changes in lifestyles, Chinese adolescents have experienced a significant decline in Physical Activity (PA) levels and a dramatic increase in Sedentary Behavior (SB). This trend is characterized by a decrease in exercise time and an increase in screen time, and is not limited to China, but is a common problem worldwide. The World Health Organization (WHO) reports that more than 80% of adolescents worldwide fail to meet its recommended daily standard of moderate to vigorous physical activity (Pierannunzio et al., 2022). Insufficient physical activity not only increases the risk of adolescents becoming overly dependent on electronic screens (Solmaz et al., 2025), it may also impair their cognitive flexibility, leading to slower responses when coping with new tasks, which can have a profound effect on physical and mental health (Li, 2015; Shang et al., 2024).

Given the important impact of executive functioning on adolescent development, it is mainly evident that executive functioning plays an important role in adolescents’ academic performance, emotional regulation, and decision-making skills (Caes et al., 2021; Pickavance et al., 2022; Quílez-Robres et al., 2021). At the same time, with advances in physiological psychology, numerous researchers have more scientific conditions and better facilities that allow them to more fully understand the relationship between physical activity and executive functioning in adolescents. Such as those conducted by Azevedo et al., have found that moderate-to-vigorous physical activity not only enhances neuroplasticity in the prefrontal cortex, but also improves an individual’s cognitive performance by increasing the secretion of brain-derived neurotrophic factor (Azevedo et al., 2020). Anggraini and Sun et al. showed that individuals with higher levels of physical activity performed better on working memory and inhibitory control tasks (Anggraini, 2023; Sun et al., 2022). On this basis, research on the mechanism of action of different types of physical activity on executive function has also received attention. For example, Benzing et al. showed that aerobic intervention can significantly improve cognitive flexibility in adolescents (Benzing et al., 2016). Deng et al. found that high-intensity interval training (HIIT), such as running, had a more significant improvement in cognitive function in adolescents (Deng and Cao, 2019). In addition to the above studies, other scholars’ studies have proved that the effect of physical activity on adolescents’ executive function is not directly manifested. For example, Zeng et al. pointed out that the effect of physical activity on adolescents’ psychology may not be directly on their executive functions, but indirectly through muscle strength (Zeng et al., 2023). Chang and Li et al. found that physical activity indirectly contributes to executive function development through cardiorespiratory endurance (Chang and Wang, 2020; Li et al., 2024).

In summary, the relationship between physical activity and adolescents’ executive function has been explored to a certain extent, but there are some problems. First, the scope of physical activity is relatively broad, and it is difficult to accurately determine the relationship between the two using “physical activity” as a variable. Second, even with the help of mediating variables, previous studies have usually used a single physical performance indicator, such as cardiorespiratory fitness and muscle strength, as a mediating factor between physical activity and executive function. However, it is difficult for a single physical fitness indicator to objectively reflect the overall physical fitness status, and it is also impossible to scientifically judge the physical activity status of an individual. And there may be synergies between different fitness indicators that affect the accuracy and interpretive power of the study results. Therefore, the present study proposes a more comprehensive measure, i.e., the Physical Fitness Index (PFI) as a mediator variable in order to integrate multiple dimensions of physical fitness qualities, such as cardiorespiratory endurance, strength, and flexibility, so as to more accurately reflect the overall fitness level of adolescents (Wang et al., 2024). Compared to a single physical fitness indicator, the Fitness Index provides a more scientific measure and helps to more objectively assess the impact of physical activity on executive function. Therefore, the present study hypothesized that the physical fitness index mediates the relationship between physical activity and adolescents’ executive functioning, aiming to provide a more scientific theoretical basis for promoting adolescents’ healthy physical and mental development.

## 2 Objects and methods

### 2.1 Objects

**Figure 1:**
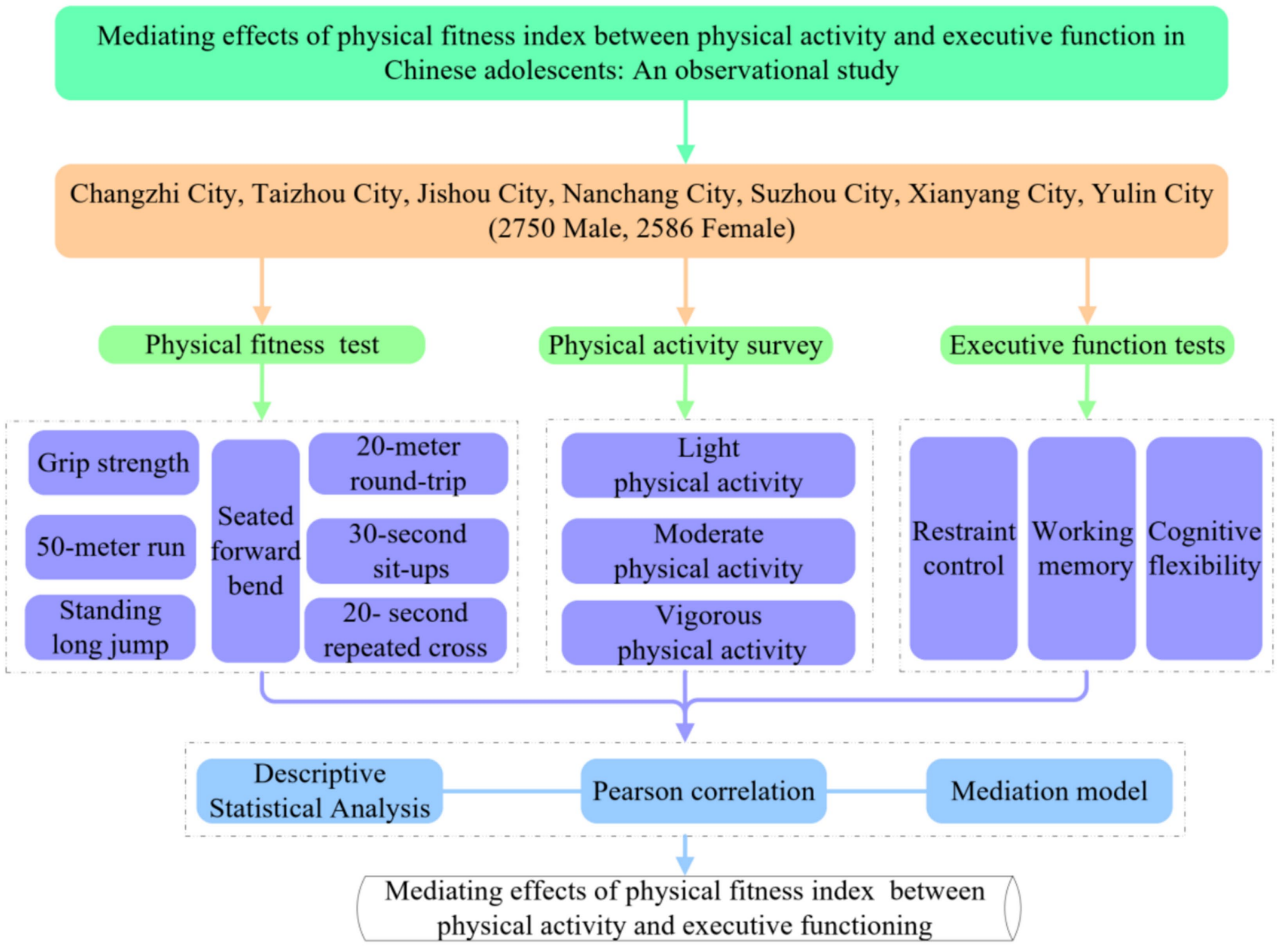
Research process.

Field tests and questionnaires were conducted from September to December 2024 in Changzhi, Taizhou, Jishou, Nanchang, Suzhou, Xianyang, and Yulin, China. Following the principle of roughly equal numbers of people of different genders and ages, a total of 18 middle schools were selected in each city, and a total of 201 classes were selected in each grade level of the middle schools using the lottery method, and all students were included in the classes. A total of 5,995 questionnaires were distributed and 5,336 valid questionnaires were recovered, with an effective recovery rate of 89%. Among them, 2,750 were male students and 2,586 were female students; the age range was 13–18 years old, 13–15 years old were junior high school students, and 16–18 years old were senior high school students. This study follows the Strengthening the Reporting of Observational Studies in Epidemiology guidelines for reporting (Vandenbroucke et al., 2007). Prior to testing, informed consent was obtained from the students and parents and the study was approved by the East China Normal University Human Experimentation Ethics Committee. Informed consent was obtained from the survey respondents (Figure 1).

### 2.2 Methods

#### 2.2.1 Physical activity survey

*The Physical Activity Level Evaluation for Children and Adolescents Aged 7 to 18 Years* was used to investigate the physical activity of the adolescents, including the program, frequency, time, and subjective perceived intensity (National bureau of disease control and prevention, 2023). In this study, physical activity was categorized into 3 intensities: low, medium, and high, where 1.5 metabolic equivalent~3 metabolic equivalent physical activity was defined as light physical activity (LPA), 3 metabolic equivalent~6 Physical activity of 3 metabolic equivalent~6 metabolic equivalent is defined as moderate physical activity (MPA). Physical activity > 6 metabolic equivalent is defined as vigorous physical activity (VPA). Total weekly physical activity time was calculated as: weekly physical activity time = number of physical activities per week × average length of each session. The questionnaire had a retest coefficient of 0.606 and a correlation coefficient of 0.689, both with a *p*-value of <0.01, giving the questionnaire good reliability and validity (Shang et al., 2024).

#### 2.2.2 Physical fitness tests

##### 2.2.2.1 Grip strength

Adolescents are holding an electronic grip strength meter (AC3249) with their hands facing outwards, standing with their feet side by side and their arms hanging down naturally. Grip twice with a strong hand and take the maximum value in kilograms, retaining 2 decimals in the test.

##### 2.2.2.2 Standing long jump

The young person stands behind the starting line, feet apart in a natural position, and jumps with both feet at the same time. Measure the distance between the back edge of the starting line and the back edge of the nearest point of impact. Each person jumps three times, and the best performance is recorded in meters with two decimals.

##### 2.2.2.3 50-m run

The youngsters stand in front of the starting line, start immediately when they hear the command of the starter, and run to the finish line with all their strength. Timing is stopped when the subject’s chest or shoulder reaches the vertical plane of the finish line, and the completed time is the final score in seconds with 2 decimals.

##### 2.2.2.4 Seated forward bend

Adolescents sat on a flat floor with their hips, back, shoulders and brain close to the wall, naturally straightening their arms, placing their hands palms down on the board of the test instrument, and straightening their legs in stirrups. During the test, the arms were stretched forward as far as possible to push the instrument until it was impossible to push the test instrument forward, in centimeters, retaining 2 decimals.

##### 2.2.2.5 20 s repeated cross

Young people stand on both sides of the center line, hear the “start” signal, step to the left or right, so that the feet were across the finish line, and then return to the center line to form the starting position, in the same way step to the other side of the finish line, and then return to the original position of the center line. Repeat this cycle, counting each cycle as four completed movements and recording the number of 20-s crossings.

##### 2.2.2.6 30-s sit-ups

Adolescents lay on a mat with their entire body on their backs, knees bent at 90 degrees, fingers crossed behind the head, and a partner pressing on the subject’s ankles. When the subject sat up, he or she touched or exceeded the same knee with the elbow. During the test, the tester started immediately after giving the “start” command and turned on a stopwatch to record the number of times the test was completed in 30 s.

##### 2.2.2.7 20-meter round trip

The 20-meter round trip test (SRT) uses the PACER test developed by Cooper Laboratories in the United States. The tester runs back and forth between two lines 20 meters apart, and must complete a slow-to-fast run (one level of speed increment per minute) according to the rhythm of the music. The test is terminated when the tester stops at exhaustion, or fails to reach the end line twice in a row before the music starts. Their performance is recorded at this point. Each 20-meter round trip is counted as 1 laps, and the final score is based on the total number of round trips completed.

The researchers standardized the seven test scores to calculate *Z*-scores, where the formula for calculating *Z*-scores is *Z* = (measured value of each physical fitness index–mean value of the index)/standard deviation. The *Z*-scores for each physical fitness index were summed to obtain the physical fitness index. In calculating the physical fitness index, the *Z*-scores for each physical fitness indicator were given the same weight, and to ensure that the physical fitness index scores were interpreted in the same direction, the researchers took the opposite number of *Z*-scores for the running test, i.e., the lower the *Z*-scores, the higher the tester’s running performance. The validity of this test method has been supported by several studies (Gao, 2022; He et al., 2021). In order to evaluate the physical fitness index more accurately and objectively, this study used the *Z* score corresponding to P_75_ as the cut-off point, and stratified the physical fitness index into two grades: *Z* scores greater than or equal to P_75_ for high grades, and *Z* scores less than P_75_ for low grades (Koren et al., 2019).

#### 2.2.3 Executive function tests

Executive function testing is performed in a quiet, distraction-free laboratory environment where all subjects are tested under the same conditions to ensure the accuracy of the data. The test tasks included an inhibitory control task, a working memory task, and a cognitive flexibility task designed by Flanker. Subjects were required to complete all tasks on a computer, and the test program was prepared by E-prime 1.1 software system (Psychology Software Tools Inc., Pittsburgh, PA, USA). Prior to the start of the experiment, subjects first received standardized instructions and practice to familiarize themselves with the task requirements. During the formal test, subjects were required to complete the task as quickly and accurately as possible, and the system automatically recorded the reaction time and the percentage of correct responses. Ultimately, the average reaction time for correct responses was used as an indicator of executive functioning, with shorter reaction times indicating better executive functioning (Wang et al., 2024).

##### 2.2.3.1 Inhibition control

In the inhibition control test, two test conditions were used, a congruent condition and an incongruent condition. First, participants were required to gaze at the center of the screen for 500 ms, after which a string of five capital letters was displayed on the screen for 1,000 ms. The two conditions, the congruent condition (e.g., LLLLL or FFFFFF) and the incongruent condition (e.g., LLFLL or FFLFF), appeared randomly during the test. Participants were asked to respond by pressing the “F” or “L” key with their left or right index finger. The test consists of a practice phase, a formal phase 1 and a formal phase 2. The practice phase consisted of 12 trials, and the formal phase I and formal phase II each consisted of 48 trials. Inhibitory control was assessed by calculating the difference between the response to the incongruent condition and the response to the congruent condition.

##### 2.2.3.2 Working memory

In the 1-back task, five capital letters such as (A, S, P, G, T) are displayed in the center of the screen. Participants were required to respond quickly based on whether the current letter was the same as the previous letter. If the letters are the same, the “F” key is pressed; if they are different, the “J” key is pressed. The task was divided into two phases, each of which was repeated 25 times. The stimulus interval for uppercase letters was 3 s, and the letters stayed on the screen for 2000 ms. In the 2-back task, participants were asked to determine whether the current letter was the same as the previous two letters and to press the “F” or “J” key accordingly. Except for the above differences, all other test requirements and methods were the same as in the 1-back task.

##### 2.2.3.3 Cognitive flexibility

The test was divided into three parts. In the first part, participants were asked to judge the size of a series of randomly appearing black numbers (1–4 and 6–9) by pressing the “D” key for less than “5” and the “F” key for more than “5.” Press “F” for greater than “5.” The average reaction time for each person was the result of their trial. In the second part, participants pressed the “J” key (odd) and the “K” key (even) according to the parity of the green numbers (1–4 and 6–9). The average reaction time for each person was their performance in all tasks. The first and second parts constituted the heterogeneous condition. In the third part, the two tasks were randomly interleaved. When a black number appeared, determine if it was less than 5 (press “D” for less than, “F” for more than, both left-handed); when a green number appeared, press “J” for odd numbers, “K” for even numbers. When a green number appears, press “J” for odd numbers and “K” for even numbers. The participant’s final score was the average reaction time (i.e., homogeneous reaction time) for all trials. The test consisted of six phases: A, B, C, C, B, A. A represented 16 trials for size, B represented 16 trials for parity, and C represented 32 trials for staggered size and parity. Practice rounds, which do not count toward the score, are performed 8 times before Part I and Part II and 16 times before Part III, respectively.

### 2.3 Mathematical and statistical methods

This study used Statistical Package for the Social Sciences (version 27.0) software for data entry and analysis. The *U* test was used to analyze the physical fitness index and physical activity characteristics of adolescents; the *t* test was used to analyze the executive function characteristics of adolescents; Pearson’s correlation was used to analyze the correlation between the study variables and based on this, the test of mediation was conducted. The mediated effects model was tested using model 4 from The PROCESS macro for R (version 4.3) prepared by Hayes, with physical activity as the independent variable, physical fitness index as the mediator variable, each of the subfunctions of executive functioning as the dependent variable, and age, gender, socioeconomic status, and screen time as control variables.

## 3 Results

### 3.1 Basic characteristics of physical activity, physical fitness index and executive function in adolescents

According to Table 1, Among middle school students, the duration of participation in moderate physical activity was 130 (72, 213) min/week in the high fitness index group, which was significantly higher than that of the low fitness index group at 109 (59, 204) min/week (*Z* = −3.598, *p* = 0.001). Among senior high school students, the duration of participation in moderate-to-vigorous physical activity was 86 (48, 143) min/week in the high fitness index group and 71 (45, 123) min/week in the low fitness index group, which was statistically significantly different when comparing the two groups (*Z* = −2.556, *p* = 0.011). Gender analysis showed that the duration of participation in moderate-to-vigorous physical activity was 133 (74, 220) min/week in the male high fitness index group and 114 (64, 211) min/week in the low fitness index group, with a statistically significant difference between the two groups (*Z* = −2.292, *p* = 0.022), and 92 (53, 158) min/week in the female high fitness index group and 75 (45, 129) min/week in the low fitness index group, with a statistically significant difference between the two groups (*Z* = −3.782, *p* = 0.001). 158 min/week in the female high fitness index group and 75 (45, 129) min/week in the low fitness index group, with a statistically significant difference between the groups (*Z* = −3.782, *p* = 0.001). Overall, the duration of participation in moderate physical activity was 110 (63, 187) min/week in the high fitness index group, which was significantly higher than 93 (53, 168) min/week in the low fitness index group (*Z* = −4.286, *p* = 0.001).

**Table 1:** Comparison of physical activity hours among adolescents with different fitness index levels [M (P_25_,P_75_), min/week].

Segments	Physical fitness index	Number	Statistical value	Light physical activity	Moderate physical activity	Vigorous physical activity	Moderate-to-vigorous physical activity
Junior high school	High grade	732		250 (140,440)	444 (250,770)	409 (220,681)	130 (72,213)
	Low grade	2,348		270 (150,510)	373 (200,710)	320 (164,630)	109 (59,204)
			Z	−2.219	−4.442	−4.057	−3.598
			P	0.026	0.001	0.001	0.001
Senior high school	High grade	569		230 (120,390)	296 (160,486)	274 (130,460)	86 (48,143)
	Low grade	1,687		210 (120,350)	247 (145,416)	210 (120,368)	71 (45,123)
			Z	−1.790	−3.492	−3.781	−2.556
			P	0.073	0.001	0.001	0.011
Male	High grade	669		250 (140,443)	418 (235,725)	421 (236,688)	133 (74,220)
	Low grade	2081		265 (135,480)	355 (190,672)	355 (190,660)	114 (64,211)
			Z	−0.553	−3.047	−3.516	−2.292
			P	0.580	0.002	0.001	0.022
Female	High grade	632		230 (120,390)	330 (172,570)	270 (140,520)	92 (53,158)
	Low grade	1954		230 (125,400)	270 (150,468)	209 (114,380)	75 (45,129)
			Z	−0.496	−4.668	−3.918	−3.782
			P	0.620	0.001	0.001	0.001
Total	High grade	1,301		240 (130,420)	360 (209,653)	345 (187,604)	110 (63,187)
	Low grade	4,035		240 (130,430)	313 (170,571)	270 (142,510)	93 (53,168)
			Z	−0.704	−5.580	−5.278	−4.286
			P	0.481	0.001	0.001	0.001

According to Table 2, adolescents’ executive functions showed some differences across gender and age groups. In the inhibitory control task, the difference between male (4.83 ± 23.23) and female (4.17 ± 23.72) was not significant (*t* = 1.017, *p* = 0.309). In the 1-back task, the mean reaction time was 831.06 ms for male and 831.92 ms for female, again with a non-significant gender difference (*t* = −0.107, *p* = 0.915). However, in the 2-back task, the mean reaction time of male (1015.79 ± 374.67) was slightly better than that of female (1035.49 ± 380.27), with the difference approaching the level of significance (*t* = −1.905, *p* = 0.057), with a significant difference in the 14-year-old (*t* = −2.358, *p* = 0.019) and 15-year-old (*t* = −3.085, *p* = 0.002) male performed significantly better than female. The most significant difference was found in the cognitive flexibility task where female (321.11 ± 142.79) significantly outperformed male (291.12 ± 137.09) (*t* = −7.816, *p* = 0.001).

**Table 2:** Basic characteristics of executive functioning in adolescents.

Age	Gender	Number	Inhibitory control	t	P	1-back	t	P	2-back	t	P	Cognitive flexibility	t	P
13	Male	426	2.84 ± 24.25	−0.101	0.920	796.57 ± 294.45	−1.210	0.226	983.77 ± 372.97	−0.931	0.352	293.63 ± 144.30	−2.507	0.012
	Female	417	3.01 ± 25.09			822.26 ± 321.46			1008.23 ± 389.98			319.45 ± 154.55		
14	Male	623	5.00 ± 24.44	1.086	0.278	814.28 ± 314.04	−0.430	0.667	1009.15 ± 386.28	−2.358	0.019	288.03 ± 148.20	−5.345	0.001
	Female	579	3.44 ± 25.38			821.86 ± 294.98			1060.46 ± 366.60			334.13 ± 150.71		
15	Male	430	4.60 ± 25.95	0.921	0.357	846.81 ± 315.47	−1.124	0.261	1013.29 ± 365.81	−3.085	0.002	292.57 ± 146.67	−4.19	0.001
	Female	408	2.99 ± 24.46			870.90 ± 304.26			1088.55 ± 338.94			333.01 ± 131.83		
16	Male	387	4.87 ± 22.59	0.001	0.999	874.52 ± 289.58	2.105	0.036	1099.97 ± 306.36	−0.137	0.891	304.98 ± 116.77	−3.823	0.001
	Female	397	4.87 ± 22.04			832.02 ± 275.70			1103.03 ± 316.82			337.40 ± 120.56		
17	Male	462	6.14 ± 20.07	0.405	0.686	833.35 ± 272.22	1.032	0.302	990.07 ± 390.91	1.598	0.110	283.32 ± 130.33	−0.74	0.459
	Female	448	5.58 ± 21.80			814.28 ± 285.19			946.74 ± 426.72			290.02 ± 142.41		
18	Male	422	5.35 ± 21.15	−0.177	0.860	832.21 ± 269.16	−0.258	0.797	1011.44 ± 396.99	0.370	0.712	287.49 ± 126.25	−2.138	0.033
	Female	337	5.63 ± 22.43			837.30 ± 272.25			1000.52 ± 413.26			308.51 ± 144.30		
Collectivity	Male	2,750	4.83 ± 23.23	1.017	0.309	831.06 ± 295.13	−0.107	0.915	1015.79 ± 374.67	−1.905	0.057	291.12 ± 137.09	−7.816	0.001
	Female	2,586	4.17 ± 23.72			831.92 ± 293.83			1035.49 ± 380.27			321.11 ± 142.79		

### 3.2 Correlation analysis of physical activity, physical fitness index and executive function in adolescents

According to Table 3, there was a significant positive correlation between vigorous physical activity, moderate physical activity, Moderate-to-vigorous physical activity and physical fitness index, with correlation coefficients of 0.044, 0.041 and 0.039 (all *p-*values <0.05). There was a significant negative correlation between physical fitness index and 1-back, 2-back and cognitive flexibility reaction times in executive function, with correlation coefficients of −0.124, −0.180 and - 0.100 (all p values < 0.01), indicating that the higher the physical activity index, the shorter the reaction time for working memory and cognitive flexibility. However, none of the correlation coefficients between the physical activity and executive function indices reached significant levels (*p* > 0.05).

**Table 3:** Correlations between physical activity, physical fitness index and executive function in adolescents.

Variant	Light physical activity	Vigorous physical activity	Moderate physical activity	Moderate-to-vigorous physical activity	Physical fitness index	1-back	2-back	Inhibitory control
Vigorous physical activity	0.391^**^							
Moderate physical activity	0.398^**^	0.986^**^						
Moderate-to-vigorous physical activity	0.392^**^	0.996^**^	0.997^**^					
Physical fitness index	0.002	0.044^*^	0.041^**^	0.039^*^				
1-back	0.004	0.011	0.005	0.014	−0.124^**^			
2-back	−0.001	0.004	−0.002	0.008	−0.180^**^	0.474^**^		
Inhibitory control	0.008	0.017	0.014	0.013	0.011	−0.013	0.007	
Cognitive flexibility	0.005	−0.02	−0.017	−0.022	−0.100^**^	0.167^**^	0.405^**^	0.001

*P < 0.05, **P < 0.01.

### 3.3 Interaction between physical activity and executive function in adolescents’ fitness indices

According to Table 4, physical activity had a significant positive effect on physical fitness index (*t* = 2.286, *p* = 0.022). Physical fitness index showed significant negative effect on working memory response time (1-back, 2-back) both (*t* = −7.519 and −11.182, *p* = 0.001, respectively), similarly physical fitness index had significant negative effect on cognitive flexibility response time (*t* = −7.210, *p* = 0.001). However, the direct effect of physical fitness index on inhibitory control did not reach a significant level (*t* = 0.949, *p* = 0.343).

**Table 4:** Interaction of body mass index between moderate-to-vigorous physical activity and executive functions.

Model	Antecedent		Coef	SE	t	*p*		Coef	SE	t	p
			Physical fitness index (M)					Inhibitory control (Y)			
Model 1	Moderate-to-vigorous physical activity (X)	a	0.000	0.000	2.286	0.022	c	0.000	0.000	0.799	0.425
	Physical fitness index (M)						b	0.110	0.116	0.949	0.343
	Constant	iY	−0.064	0.070	−0.910	0.363	iY	4.876	0.462	10.543	0.001
Model 2			Physical fitness index (M)					1-back (Y)			
	Moderate-to-vigorous physical activity (X)	a	0.000	0.000	2.286	0.022	c	0.003	0.002	1.095	0.274
	Physical fitness index (M)						b	−11.069	1.472	−7.519	0.001
	Constant	iY	−0.064	0.070	−0.910	0.363	iY	829.334	5.847	141.843	0.001
Model 3			Physical fitness index (M)					2-back (Y)			
	Moderate-to-vigorous physical activity (X)	a	0.000	0.000	2.286	0.022	c	0.002	0.003	0.823	0.411
	Physical fitness index (M)						b	−20.676	1.849	−11.182	0.001
	Constant	iY	−0.064	0.070	−0.910	0.363	iY	1014.186	7.343	138.113	0.001
Model 4			Physical fitness index (M)					Cognitive flexibility (Y)			
	Moderate-to-vigorous physical activity (X)	a	0.000	0.000	2.286	0.022	c	−0.001	0.001	−0.581	0.561
	Physical fitness index (M)						b	−5.043	0.699	−7.210	0.001
	Constant	iY	−0.064	0.070	−0.910	0.363	iY	303.829	2.778	109.375	0.001

This study used mediation effects analysis to investigate the mediating role of adolescent physical fitness index between moderate-to-vigorous physical activity and executive functioning, controlling for confounding variables such as age, gender, socioeconomic status, and screen time. According to Table 5, the total (*β* = 0.0002, *p* = 0.3337) and direct (*β* = 0.0002, *p* = 0.3519) effects did not reach the level of significance in the inhibitory control task, and the indirect effect was 0 (95% CI: 0.0000–0.0000), suggesting a non-mediated effect. In the 1-back task, the indirect effect was −0.0007 (95% CI:-0.0021 - -0.0003), accounting for 31.82% of the total effect. The indirect effect of the 2-back task (−0.0013, 95% CI:-0.0039--0.0007), which accounted for 100.0% of the total effect, suggests that physical fitness index fully mediated physical activity on 2-back performance. The indirect effect of cognitive flexibility (−0.0004, 95% CI:-0.0010--0.0002) accounted for 66.67% of the total effect.

**Table 5:** Mediating effects of adolescent fitness index between physical activity and executive functioning.

Executive function	Group	Effect	SE	t	p	LLCI	ULCI
Inhibitory control	Total effect	0.0002	0.0002	0.9669	0.3337	−0.0002	0.0006
	Direct effect	0.0002	0.0002	0.9310	0.3519	−0.0002	0.0005
	Indirect effect	0.0000	0.0000	/	/	0.0000	0.0000
1-back	Total effect	0.0022	0.0024	0.9250	0.3550	−0.0025	0.0069
	Direct effect	0.0029	0.0024	1.2346	0.2171	−0.0017	0.0076
	Indirect effect	−0.0007	0.0005	/	/	−0.0021	−0.0003
2-back	Total effect	0.0013	0.0030	0.4412	0.6591	−0.0046	0.0072
	Direct effect	0.0027	0.0030	0.9000	0.3682	−0.0031	0.0085
	Indirect effect	−0.0013	0.0009	/	/	−0.0039	−0.0007
Cognitive flexibility	Total effect	−0.0006	0.0011	−0.5520	0.5810	−0.0028	0.0016
	Direct effect	−0.0003	0.0011	−0.2360	0.8134	−0.0025	0.0019
	Indirect effect	−0.0004	0.0002	/	/	−0.0010	−0.0002

This model controls for age, gender, socioeconomic status, and screen time.

## 4 Discussion

In the present study, it was found that physical activity was positively correlated with physical fitness index in adolescents, i.e., the longer the duration of physical activity the higher the physical fitness index. The present results are generally consistent with previous studies. For example, He et al. showed a significant positive correlation between moderate-to-vigorous physical activity and physical fitness index (He et al., 2021). Gao’s study also showed that the longer the time spent in daily physical activity, the higher the physical fitness index (Gao, 2022). The results of this study further support the role of physical activity in promoting physical fitness development in adolescents. The positive correlation between physical activity and physical fitness index may stem from multiple mechanisms. First, physical activity improves the physical fitness index by increasing energy expenditure and promoting the metabolism of fats and sugars. It has been shown that the longer the duration of daily physical activity, the higher the physical fitness index, which further confirms the positive effect of physical activity on physical fitness (Li, 2022). Second, physical activity enhances muscular strength and cardiorespiratory endurance. For example, aerobic interventions can increase stride speed and cardiorespiratory fitness levels, while high-intensity exercise (e.g., jumping training) can enhance the utilization of elastic potential energy of the muscles, increase explosive power and reaction speed, and thus further improve the physical fitness index (Ma et al., 2022; Ng et al., 2020). Studies have found that males outperform females in cognitive flexibility. Gender differences may stem from the interaction of physiological and psychological factors. It has been shown that men typically exhibit higher strength and endurance during exercise, which may contribute to higher body mass index and further impact executive function (Liu et al., 2021). In addition, differences in hormone levels (e.g., testosterone and estrogen) may also play a role in the development of executive function (Schneider et al., 2017).

The present study also found that physical activity indirectly contributes to the development of executive function through the mediating role of the physical fitness index. Although the mediating effect values were low, they had an important impact on adolescents’ cognitive development. For example, regular physical activity over a long period of time consistently enhances cardiorespiratory fitness and promotes the development of executive functions by improving cerebral blood flow supply (Wang et al., 2023). However, no direct correlation was found between physical activity and executive function, a phenomenon that may be due to a number of factors. First, the sensitivity of the measurement instrument may be a key factor influencing the results. Since the present study was based on an experimental task approach to measuring executive function, it may not be sensitive enough to capture the weak effects of physical activity on executive function. Second, external factors (e.g., screen time, stress levels) may interfere with executive function. For example, it has been noted that excessive screen use in adolescents reduces executive function performance (Moshel et al., 2024). Chronic academic stress may also affect their cognitive abilities (Zhang et al., 2019). Inconsistency between the results of this study and some previous studies (Siersbaek et al., 2022; Williams et al., 2022). Possibly due to differences in sample characteristics and research methods, firstly, samples from different studies may differ in terms of age, sex ratio and physical activity level. For example, the participants in the Sánchez study were predominantly athletes, whereas the sample in the present study consisted of general adolescents, which may have led to different effects of physical activity on executive function (Sánchez García et al., 2024). Second, differences in research methodology may also be an important factor contributing to inconsistent results. For example, Tuvey used a longer physical activity intervention and incorporated more control variables in the experimental design (Tuvey et al., 2019), whereas the present study was based on cross-sectional data, which did not allow for direct inferences of causality.

In summary, the strengths of this study include the use of a large sample size and the combination of objective measures and subjective reports assessing physical activity, physical fitness indices, and executive functioning, but some limitations remain. This is mainly reflected in the following 3 points: (1) the cross-sectional design limits the inference of causality; (2) physical activity was measured mainly by self-report, which may be subject to recall bias; and (3) the present study did not address the assessment of executive functioning in dual-task contexts, which fails to comprehensively reveal the effects of physical activity on executive functioning. Future studies could use longitudinal tracking studies to delve deeper into the causal relationship between physical activity and executive function, as well as combining objective measurements with wearable devices (e.g., accelerometers, heart rate monitors) to improve the accuracy and reliability of the test data, and introducing dual-tasking experiments for a more refined assessment of the effects of physical activity on different dimensions of executive function (Deodato et al., 2024; Longhini et al., 2024). Based on the findings of this study, it is recommended that schools and families should provide adolescents with more opportunities to engage in moderate-to-vigorous physical activity and implement targeted training based on individual fitness differences to optimize their fitness development and executive function enhancement. Meanwhile, physical education programs can incorporate digital monitoring tools to more effectively assess the long-term effects of physical activity on individual health and executive function.

## 5 Conclusion

In this study, we found that there was a significant positive correlation between moderate-to-vigorous physical activity and physical fitness index in adolescents; there was a significant negative correlation between physical fitness index and working memory (1-back, 2-back) and cognitive flexibility reaction times; and there was no significant correlation between physical fitness index and inhibitory control reaction times. And there was a fully mediated effect of physical fitness index between moderate-to-vigorous physical activity and executive function.

## Data Availability

The original contributions presented in the study are included in the article/supplementary material, further inquiries can be directed to the corresponding author.
